# Effect of Penthorum Chinense Pursh Compound on AFB1-Induced Immune Imbalance via JAK/STAT Signaling Pathway in Spleen of Broiler Chicken

**DOI:** 10.3390/vetsci10080521

**Published:** 2023-08-13

**Authors:** Qin Lu, Yu Hu, Fazul Nabi, Zhenzhen Li, Habibullah Janyaro, Wenyan Zhu, Juan Liu

**Affiliations:** 1 Immunology Research Center of Medical Research Institute, Southwest University, Chongqing 402460, China; lqindz@163.com; 2 College of Veterinary Medicine, Southwest University, Chongqing 402460, China; 18783648042@163.com (Y.H.); fazulnabishar@yahoo.com (F.N.); lizhenzhenaaa0125@163.com (Z.L.); 3 Wanzhou District Livestock Industry Development Center, Chongqing 404020, China; 4 College of Animal Science and Technology, Chongqing Three Gorges Vocational College, Chongqing 404155, China; 5 Department of Veterinary Surgery, Shaheed Benazir Bhutto University of Veterinary and Animal Science, Sakrand 67210, Pakistan; janyaroh@gmail.com; 6 College of Pharmacy, Chongqing Medical University, Chongqing 400016, China; 7 Chongqing Engineering Research Center of Pharmaceutical Sciences, Chongqing Medical and Pharmaceutical College, Chongqing 401331, China

**Keywords:** *Penthorum chinense* Pursh Compound, Broiler chicken, AFB1, JAK/STAT signaling pathway, apoptosis, immune imbalance

## Abstract

**Simple Summary:**

Aflatoxin B1(AFB1) is the main secondary metabolite produced by Aspergillus flavus, which is highly toxic, carcinogenic, mutagenic and teratogenic. It can induce immune imbalance in animals or humans. *Penthorum chinense* Pursh (PCP) is a traditional herbal plant that has been used as a hepatoprotective drug with a long history in China. Based on the theory of traditional Chinese Medicine, we prepared *Penthorum chinense* Pursh Compound (PCPC) by combining four herbal medicines: *Penthorum chinense* Pursh, *Radix bupleuri*, *Artemisia capillaris Thunb* and *Radix glycyrrhizae*. The role of the *Penthorum chinense* Pursh Compound (PCPC) in preventing AFB1-induced immune imbalance in broiler chickens was studied. The results showed that PCPC attenuated AFB1-induced spleen damage and alleviated the imbalance of pro-inflammatory and anti-inflammatory cytokines, counteracting AFB1’s capacity to promote apoptosis. PCPC’s protective effects in AFB1-associated spleen injury may be through the JAK/STAT pathway. The above results indicate that PCPC can be used as a safe and effective drug to inhibit inflammation and prevent the AFB1-induced spleen apoptosis via JAK/STAT pathway in the spleen.

**Abstract:**

Aflatoxin B1(AFB1) is the main secondary metabolite produced by Aspergillus flavus, which is highly toxic, carcinogenic, mutagenic and teratogenic. It can induce immune imbalance in animals or humans. *Penthorum chinense* Pursh (PCP) is a traditional herbal plant that has been used as a hepatoprotective drug with a long history in China. Based on the theory of traditional Chinese Medicine, we prepared *Penthorum chinense* Pursh Compound (PCPC) by combining four herbal medicines: 5 g *Penthorum chinense* Pursh, 5 g *Radix bupleuri*, 1 g *Artemisia capillaris Thunb* and 1 g *Radix glycyrrhizae*. The role of the *Penthorum chinense* Pursh Compound (PCPC) in preventing AFB1-induced immune imbalance in broiler chickens was studied. A total of 180 broiler chickens were equally distributed in six groups: controls, AFB1, YCHD and high-, medium- and low-dose PCPC treatment groups. After 28 days, broilers were anesthetized, and serum spleen and thymus samples were collected for analysis. Results show that AFB1 significantly increased and decreased the relative organ weight of the spleen and thymus, respectively. Pathological section of hematoxylin/eosin (H&E) stained spleen sections showed that AFB1 resulted in splenic tissue damage. Both the serum levels of Immunoglobulin A (IgA) and Immunoglobulin G (IgG) were suppressed in the AFB1 group. IL-6 was elevated in the AFB1 group. The balance between pro-inflammatory cytokines (IFN-γ and IL-2) and anti-inflammatory cytokine (IL-4) was disturbed by AFB1. The apoptosis-related protein and JAK/STAT pathway-related gene expression indicated that AFB1-induced apoptosis via JAK/STAT pathway. PCPC has proven its immunoprotective effects by preventing AFB1-induced immune imbalance. PCPC can be applied as a novel immune-modulating medicine in broiler chickens. It can be applied as a novel immune modulator in veterinary clinical practice.

## 1. Introduction

Aflatoxins are metabolite products of Aspergillus flavus and Aspergillus parasiticus under certain growing and storage conditions. Aflatoxin B1(AFB1) is the most toxic mycotoxin, which can cause multiple organ damage, decreased egg production rates and growth retardation in poultry [1,2,3]. Additionally, aflatoxin residues in livestock products might transmit to humans via the consumption of livestock and dairy products and transmitted a serious risk to human health [4]. 

Among its diverse harms, its inhibitory role in the immune system has drawn extensive attention over recent years [5]. Research has demonstrated that AFB1 may interfere with cell cycle progression and apoptosis, leading to histopathological damage in the thymus and bursa of poultry [6]. Studies have also shown that AFB1-induced mitochondria-directed apoptosis of mouse spleen that is correlated with increased oxidative stress [7]. The spleen is the animal’s main peripheral immune organ and plays an important role in both innate and adaptive immunity. The spleen is the largest organ of the body lymphatic system, it plays an important role in the modulation of the immune system via the clearance of circulating apoptotic cells, the differentiation and the activation of T and B cells [8]. AFB1 may cause an M1/M2 imbalance by switching macrophage polarization towards a pro-inflammatory M1 phenotype [9]. AFB1 may also cause the over expressions of CD3^+^ and CD8^+^ T cells and results in the down regulations of the expression of anti-inflammatory cytokines IL-4, although an increase in pro-inflammatory cytokines IFN-γ, TNF-α and IL-6 [10,11]. Recent studies have shown that methylation of DNA, modification of RNA and modification of histones are involved in regulating the toxicity of mycotoxins [12]. AFB1 exposure decreased the phagocytic capacity of macrophages by upregulating the expression of DNA methyltransferases (DNMT1 and 3a) [13]. Ultimately, this leads to immune dysfunction, cytokine imbalances, immune response suppression, vaccine failure and disease susceptibility [10,14,15,16,17]. Although the liver is the primary target organ for aflatoxins, the aflatoxin-induced imbalance in the inflammatory immune response is also noteworthy and requires interventions.

During the animal and cell model experiments, the ROS burst was observed in these models of AFB1 exposure, and STAT3 and STAT5A were known for their role in inhibiting ROS bursts [18,19]. AFB1 exposure may down-regulated the STAT3 and p-STAT3 Ser727 proteins in macrophages. Janus kinase/signal for transcription (JAK/STAT) transducers and activators play a main part in regulating apoptosis, proliferation, differentiation and immune response [20,21]. JAK/STAT pathway is essential for induction of innate and adaptive immunity, and ultimately suppressing inflammatory and immune responses [22,23]. In this regard, JAK/STAT pathway may hold an important role in the immune imbalance induced by AFB1.

Scientists are investigating natural drugs because they are cost-effective and have fewer side effects [24,25]. *Penthorum chinense* Pursh, a traditional protective drug for hepatitis, has antioxidant and anti-inflammatory properties and has long been used to treat jaundice, edema and viral hepatitis [26]. *Penthorum chinense* Pursh contain a wide variety of chemical components comprising flavonoids, organic acids, coumarins, lignans, polyphenols and sterols, which show numerous biological effects including antidiabetic, anti-inflammatory and immune-modulation activities [27,28]. *Penthorum chinense* Pursh Compound (PCPC) is composed of four herbal ingredients: 5 g of *Penthorum chinense* Pursh, 5 g of *Radix bupleuri*, 1 g of *Artemisia capillaris* Thunb and 1 g of *Radix glycyrrhizae*. PCPC contains a unique set of flavonoids, such as gallic acid, protocatechuic acid, rutin, liquiritin, quercetin, isorhamnetin and kaempferol.

Based on relevant literature and pilot experiments, we hypothesized that the JAK/STAT signaling pathway could be an important pathway by which AFB1 disturbs the immune balance. We sought to explore the impact of PCPC therapy on immune imbalanced poultry exposed to AFB1. In addition, the likely mechanisms behind this effect have been explored, in particular its effects on inflammation, apoptosis and the JAK/STAT signaling pathway.

## 2. Materials and Methods

### 2.1. Drug Preparation and AFB1 Extraction 

The Chinese medicinal plants (*Penthorum chinense* Pursh, *Radix bupleuri*, *Artemisia capillaris* Thunb, *Radix glycyrrhizae*, *Rheum officinale* Baill and *Gardenia iasminoides* Ellis) were purchased from Chongqing Renyuantang, China. *Penthorum chinense* Pursh Compound (PCPC) and Yinchenhao Decoction (YCHD) extraction were prepared according to previously published methods [29].

Aspergillus flavus (NRRL3357) was utilized in this study and obtained from the School of Life Sciences, Sun Yat-Sen University, China. Aspergillus flavus was cultured in PDA medium at 32 °C for 5 days, then inoculated with sterilized corn flour under standard conditions (32 °C, 80–90% of relative humidity) for 14 days to prepare AFB1-containing corn flour. Finally, AFB1 content was quantified by HPLC. 

### 2.2. Experimental Design, Management, Drug Treatment 

Broiler chickens (day-old, mixed) have been bought from Chengdu Muxing Poultry Co., Ltd., Sichuan, China. Prior to modelling, all broiler chickens were stored adaptively for seven days. A total of 180 broiler chickens were randomly divided into six groups (n = 30 per group): (1) Control group (basal diet) details are provided in Supplementary Table S1; (2) AFB1 group (2.8 mg/kg AFB1-containing diet); (3) YCHD group (2.8 mg/kg AFB1 and 8 g/kg YCHD-containing diet); (4) PCPC-H group (2.8 mg/kg AFB1 and 12 g/kg PCPC-containing diet); (5) PCPC-M group (2.8 mg/kg AFB1 and 8 g/kg PCPC-containing diet); and (6) PCPC-L group (2.8 mg/kg AFB1 and 4 g/kg PCPC-containing diet). Regular food and water were available ad libitum. All experiments were carried out under the supervision of the Institutional Animal Care and Use Committee (IACUC) of the University of Southwest, in compliance with animal ethics protocols and guidelines.

### 2.3. Blood and Tissue Samples Collection 

After 28 days, broilers were anesthetized with an intraperitoneal phenobarbital injection. Blood was collected and centrifuged at 3000 rpm for 8 min. Serum was stored at −80 °C until analysis. Spleen and Thymus were weighed, and Relative organ weight was calculated as an absolute organ weight (g)/body weight on sacrifice day (kg) [30]. The tissue samples were fixed in 10% formalin for histological examination of the spleen, and the other sections were stored at −80 °C and frozen with liquid nitrogen for other analysis further. 

### 2.4. Histologic Evaluation

Samples of spleen tissue were fixed in 10% formalin for 72 h and dehydrated in alcohol and xylene, incorporated into paraffin. The incorporated samples were cut into 5 μm thick sections and stained with H&E. The samples were finally sealed with neutral gum and imaged with an optical microscope, 100× objective.

### 2.5. Cytokine and Apoptosis Proteins Levels in Spleen

The frozen spleen samples were homogenized in a buffered saline phosphate solution (PBS). The expression level of cytokines, i.e., IL-2, IL-4, IL-6 and IFN-γ and apoptosis-related proteins Bcl-2, Bcl-xl, Bax and Caspase-3 were carried out using a Cell Death Detection ELISA kit (Xiamen Biological Technology Co., Ltd., Xiamen, China). The optical density (OD) at 450 nm was quantified with an immunoenzyme plate reader (ELISA).

### 2.6. Serum Immunoglobulin

All experiments were carried out as per the manufacturer’s instructions. Serum immunoglobulin A (IgA) and immunoglobulin G (IgG) levels were determined using an ELISA kit (Xiamen Huijia Biological Technology Co., Ltd., Xiamen, China).

### 2.7. Extracting RNA and qPCR

The spleen tissue was homogenized using a homogenizer. Total RNA was extracted based on the following protocol for the TRIZOL reagent (TaKaRa, Japan). Total RNA was quantified using a nanodrop spectrometer, and RNA integrity was determined using an agarose gel electrophoresis at 1.5% (*v*/*v*). Complementary DNA (cDNA) was synthesized using a Reverse Transcription Kit (TransGen Biotech, China). The relative mRNA was quantified by qPCR using SYBR Green dye. Specific primers for *Jak2*, *Jak3*, *Stat3*, *Stat5* and *Gapdh* (Table 1) were designed using Oligo 6.0 software and were synthesized by Sangon Biotech (Shanghai, China). The real-time quantitative PCR was performed using SYBR Green I in accordance with the manufacturer’s instructions in a 96-well plate on a Lightcycler96 (Roche) real-time PCR system. Relative gene expression levels were calculated using the 2 ^−ΔΔCT^ method with normalization compared to GAPDH.

### 2.8. Statistical Analysis

Statistical analyses were conducted using SPSS 22.0 (SPSS Inc., Chicago, IL, USA). The data were analyzed using one-way analysis of variance (ANOVA). Data were presented as mean ± standard deviation. *p* < 0.05 was considered statistically significant. Throughout, * *p* < 0.05, ** *p* < 0.01; not significant (ns) *p* > 0.05. Prism (GraphPad Software 8.0.1) was used to generate graphs.

## 3. Results

### 3.1. Spleen and Thymus Relative Weight

**Figure 1:**
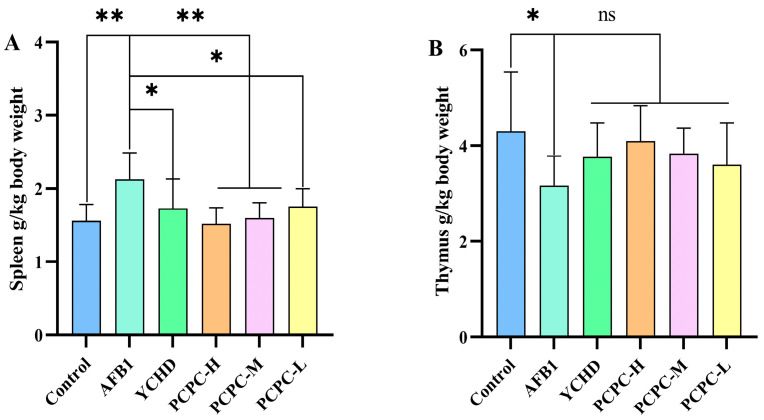
The relative weight of the spleen and thymus of the immune organs. The protective effect of PCPC on spleen and thymus in broiler chickens fed a feed containing AFB1. Results were expressed as mean ± SD. (**A**) Spleen; (**B**) Thymus. (* *p* < 0.05, ** *p* < 0.01; and non-significant (ns) *p* > 0.05).

The spleen is the body’s most important secondary immune system and increased spleen weight is used as a marker for the severity of inflammation. The thymus is defined as a primary lymphoid organ, which plays a significant role in the immune system development and differentiation. Relative organ weight was calculated as an absolute organ weight (g)/body weight on sacrifice day (g) × 100. The organs of 30 animals per group were examined. Compared with the control group, the relative organ weight of the spleen in the AFB1 group increased markedly (*p* < 0.01), while no significant enlargement was noted in all treated groups, especially in the PCPC-H group. Increased spleen weight in the AFB1 group may be associated with the expansion of the red and white pulp. The relative weight of thymus organs in the AFB1 group decreased significantly (*p* < 0.05). There was a tendency for improvement in all treatment groups, especially in the PCPC-H group (Figure 1). The relative weight of the organ thymus of the model group was significantly reduced as compared to the control group, while the relative weight of the spleen was remarkably increased, indicating that AFB1 could destroy immune organs. YCHD and PCPC, especially PCPC-H, could effectively protect the thymus and spleen from AFB1 in broiler chickens.

### 3.2. The Protective Effect of PCPC on the Damage of Spleen Tissue Structure

**Figure 2:**
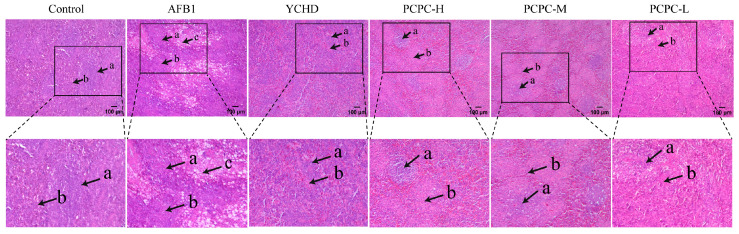
The figure shows H&E staining of the spleen of broiler chickens (100×). Letters “a” indicates white pith, “b” indicates red pith and “c” indicates cavities.

Histopathological changes in the spleen tissue were assessed by H&E staining (Figure 2). In the Control group, the spleen tissue was tightly arranged with clear boundaries between the white pith (WP) and red pith (RP), and the spleen cells were neatly arranged with no abnormal changes. In the AFB1 group, the spleen showed deformation of the red and white pith, obvious cavities, reduced and loosely arranged red and white pith and necrosis of white pith lymphocytes. The overall situation was significantly better in all drug groups, especially in the PCPC-H group, where the white pith and red pith were clearly demarcated, and some white pith had atrophy. Improved splenic histopathology in the drug group indicated that the PCPC was effective in protecting the spleen from AFB1 lesions.

### 3.3. The Protective Effect of PCPC on Serum Immunoglobin

**Figure 3:**
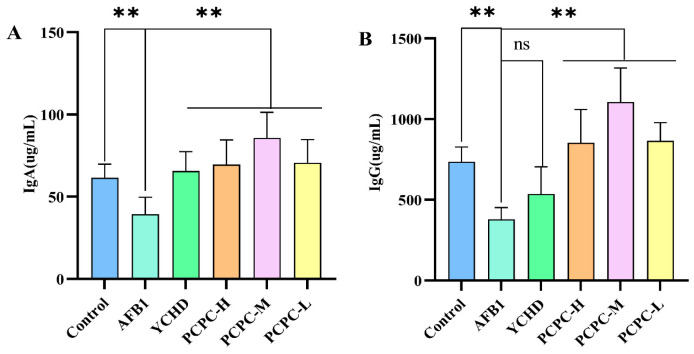
The level of serum immunoglobulin in AFB1-induced immune imbalance in broiler chickens. Results were expressed as mean ± SD. (**A**) IgA; (**B**) IgG. (** *p* < 0.01; and non-significant (ns) *p* > 0.05).

Results from the serum immunoglobin analysis are shown in Figure 3. Compared with Control, AFB1-induced immune imbalance significantly decreased the IgA and IgG levels (*p* < 0.01). The levels of IgA and IgG were significantly increased in PCPC-treated groups as compared with AFB1-induced group, (*p* < 0.01).

### 3.4. The Effect of PCPC on Cytokine Levels in Spleen

**Figure 4:**
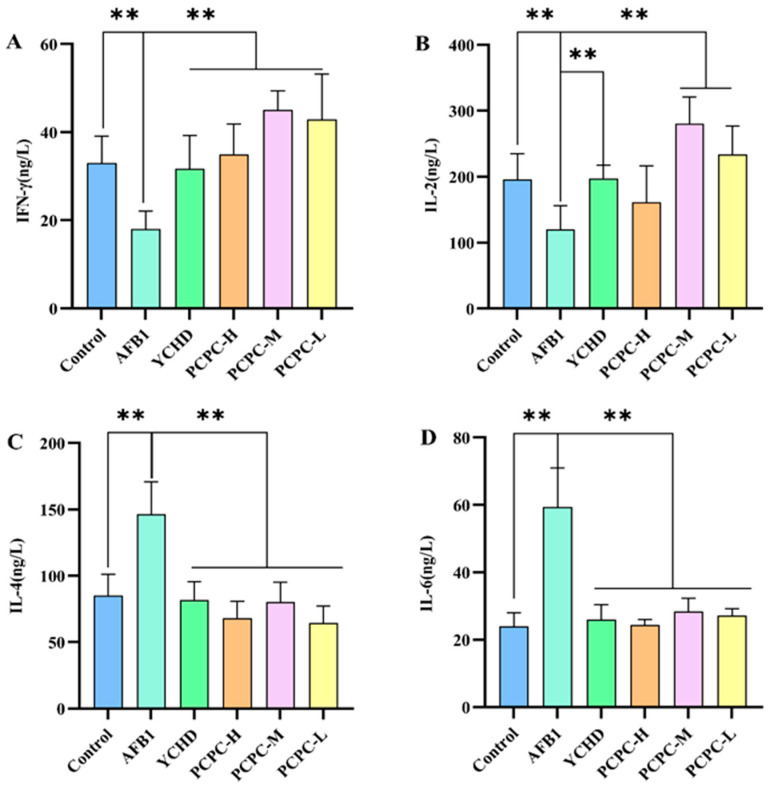
Cytokine levels in spleen of broiler chickens. Results were expressed as mean ± SD. (**A**) IFN-γ; (**B**) IL-2; (**C**) IL-4; (**D**) IL-6. (** *p* < 0.01).

Our results shown in Figure 4, increased the expression level of the inflammatory cytokine in the spleen of broiler chickens. Compared with Control, AFB1-induced immune imbalance significantly decreased the level of INF-γ and IL-2 and increased the level of IL-4 and IL-6 (*p* < 0.01). The normal cytokine levels were maintained in YCHD and PCPC-treated groups (*p* < 0.01) as compared with the AFB1 group. 

### 3.5. Effects of PCPC on the Expression of Apoptosis-Related Protein

**Figure 5:**
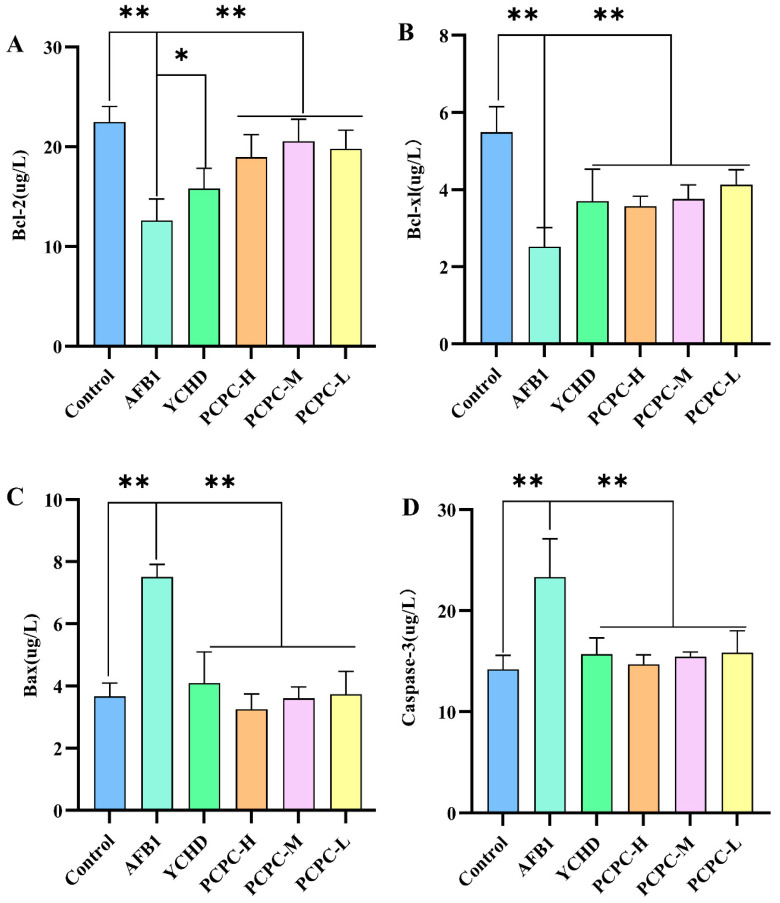
Expression of the protein connected with apoptosis. Results were expressed as mean ± SD. (**A**) Bcl-2; (**B**) Bcl-xl; (**C**) Bax; (**D**) Caspase-3. (* *p* < 0.05, ** *p* < 0.01).

Apoptotic protein expression was analyzed by ELISA (Figure 5). AFB1 significantly decreased Bcl-2 and Bcl-xl (*p* < 0.01) and increased Bax and Caspase-3 protein levels (*p* < 0.01). On the other hand, the administration of PCPC significantly suppressed the expressions of Bcl-2 and Bcl-xl and increased the expressions of Bax and Caspase-3. In general, apoptosis is controlled by pro-apoptotic (Bax) and anti-apoptotic (Bcl-2 and Bcl-xl) proteins and is carried out through Caspase (Caspase-3). It was noted that AFB1 promoted apoptosis in the spleen. PCPC has been found to modulate apoptosis via Bax/Bcl-2/Caspase-3, showing protective effects by regulating Bcl-2/Bax ratio and reducing Caspase-3 expression.

### 3.6. PCPC Inhibits AFB1-Induced Apoptosis via JAK/STAT Pathway

**Figure 6:**
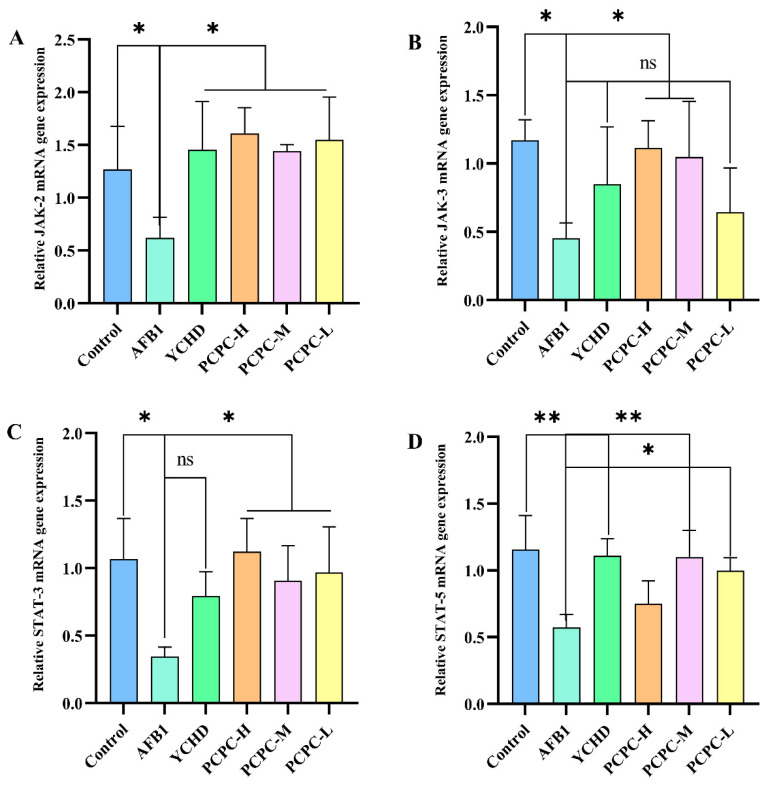
Protective effects of PCPC on apoptosis due to AFB1 by interfering with the JAK/STAT pathway. The JAK/STAT-associated gene mRNA expression was analyzed by real-time quantitative PCR (qPCR). Relative mRNA result expressed as mean ± SD. (**A**) JAK2, (**B**) JAK3, (**C**) STAT3 and (**D**) STAT5. (* *p* < 0.05, ** *p* < 0.01, non-significant (ns) *p* > 0.05).

To elucidate the signaling pathway that AFB1 promotes apoptosis in the spleen, we examined JAK2, JAK3, STAT3 and STAT5 mRNA expression in the spleen by qPCR (Figure 6). The results showed that JAK2, JAK3, STAT3 and STAT5 mRNA expression was significantly downregulated within the AFB1-fed group significantly (*p* < 0.05). Though, these changes were very minor across all treatment groups. Our findings suggest that PCPC may have protective effects on AFB1-induced apoptosis in the spleen by interfering with the JAK/STAT pathway.

## 4. Discussion

The use of Chinese traditional medicines in veterinary clinical practice has recently increased due to less toxicity. *Penthorum chinese* Pursh is a new folk medicine rich in biomolecules (flavonoids, organic acids and terpeneoids). It is important biomolecules (5-hydroxyflavanone-7-O-β-D-glucoside, quercetin, kaempferol, pinocembrin and catechins) reported various effects including hepato-protective, nephroprotective, anti-inflammatory and immune inequality in addition anti-oxidation in veterinary and human clinical applies [31]. Hepato-protective and nephron-protective properties of *Penthorum chinense* Pursh and its related phytotherapeutic compounds have already been proved in veterinary and poultry [29,32]. Aflatoxin B1 intoxication in the feed results in an immune imbalance in the broiler chicken, as their immune system is already in the early developmental process. Herein, we reported the effects of PCPC in reversing the immune imbalance in AFB1-challenged broiler chickens, and their regulation via JAK/STAT pathway [33]. The spleen is the largest and most important peripheral immune organ in the animal body. The spleen is the main reservoir of various immune cells. It does several vital jobs to keep the healthy body of animals by improving their immune function [34]. Due to this function combining the innate and adaptive immune response, the spleen is the most important organ for antibacterial and anti-fungal immune reactivity [35].

Our previous results have shown that treatment with the *Penthorum chinense* Pursh Compound protects kidney cells from excessive apoptosis via inhibiting mitochondrial apoptosis through the AFB1 pathway regulation [29]. In addition to the treatment of PCP extract of AFB-1-challenged liver toxicity was calculated and the significance of AFB1, oxidative stress and fatty degenerations were reversed by the addition of PCP extract in broiler chicken [31]. Therefore, this research was undertaken to additional study the ameliorating properties of *Penthorum chinense* Pursh Compound on AFB1-induced Immune Imbalance through the JAK/STAT signaling pathway in the Spleen of Broiler Chickens. In the present study, AFB1-challenged group spleen indexes were increased, and spleen cellular and tissue structures were damaged indicating that immune-related mechanisms were imbalanced, while PCPC reversed the spleen index and cellular/tissue structures. The enlargement of the spleen might be due to an imbalance in cytokines production and shifting with the intoxication of AFB1. Similar reversion effects of PCPC were observed in hepatic and nephritic tissues of AFB1-challenged broiler chicken [29,32]. There are two types of T cells (CD4^+^ T cells and CD8^+^ T cells) when immature CD4^+^ T cells are activated by competent antigen-presenting cells and further differentiated and finally converted to Th1 and Th2 cells [36]. Th1 cells are involved in the cellular immune responses by producing TNF-α, IFN-γ and IL-2, while Th2 cells are concerned with the production of IL-4, IL-5, IL-10 and IL-13. Th1/Th2 via cytokines production is involved in the establishment and development of T cell-derived immune responses. When an imbalance occurs in these immune-related mechanisms leading to immune system diseases, and finally immune imbalance [37,38,39], it is well established that flavonoids and traditional biomolecules play immunomodulatory roles by acting on Th1 and Th2, leading to cytokine-mediated immune responses, combat inflammation and infection, finally maintaining immune homeostasis [40,41,42,43].

In this study, IFN-γ and IL-2 expression were down-regulated, and IL-4 and IL-6 were significantly increased in AFB1 medicated group resulting in the enlargement of spleen size, which was later reversed with PCPC medication. Inflammatory cytokines shifting and secretion lead to enlargement of the spleen and activation of JAK-STAT pathways. Cytokines are a category of signaling molecules that act as a ligand to bind to cell membrane receptors. Thereby activating the JAK non-receptor tyrosine kinase associated with the cell membrane receptor, then refereeing the phosphorylation of the downstream proteins of the JAK-STAT pathways and using the corresponding immune function [44]. Activations and the expression of the JAK/STAT signal can be enhanced in many cells, giving them resistance to apoptotic stimuli [45]. The JAK/STAT signaling pathway was also found in AFB1-induced inflammation, liver fibrosis and hepatocellular carcinoma (HCC) [46,47]. The JAK/STAT pathway is involved in many important biological processes such as cell proliferation, differentiation, apoptosis and immune regulation and is universal and necessary for cytokine receptor signaling [48], associated with innate and adaptive immunity [49,50,51]. This signaling pathway is a common pathway for many cytokine signal transduction. After activating the receptor, the signal needs to pass through four JAK tyrosine kinases (JAK1, JAK2, JAK3 and Tyk2) and a combination of members of seven STAT families (STAT1, STAT2, STAT3, STAT4, STAT5a, STAT5b and STAT6) [52]. The downstream signaling of JAK-STAT is thought to be mainly performed by “mitogen-activated protein kinase” (MAPK) and “protein kinase B pathway” (PI3K/AKT). These pathways can be pathologically activated by ligand binding (under chronic inflammatory conditions) or by activating mutations within the JAK gene) [53]; There is a plentiful sign that JAK3 protein plays a significant role in lymphocyte maturation and function, and it mostly facilitates and contributes to the creation of cytokines such as IL-2 and IL-4 [54,55]; therefore, the lack or disorder of JAK3 will lead to lymphocyte dysfunction and ultimately lead to immune imbalance. Pleiotropic cytokine IL-6 is a marker of inflammatory response [56]. IFN-γ and IL-2 were pro-inflammatory cytokines, which were mainly secreted by Th1. IFN-γ is important for the clearance of intracellular pathogens [57]. IL-4 was an anti-inflammatory cytokine, which was mainly secreted by Th2. The balance between Th1 and Th2 plays an important role in immune responses in multiple diseases [58,59,60].

Studies have shown that JAK3 may be a more attractive target because it exhibits the greatest immune imbalance effect and has the most profound impact on the treatment of inflammatory diseases. In recent years, the JAKs family has been identified as an attractive therapeutic target for inflammatory diseases in the biotechnology and pharmaceutical fields [61]. Apoptosis is programmed cell death related to healthier immunity of the organism [62]. It is well established that Bcl-2 and Bcl-xl are regulating the apoptosis genes and in a downstream way regulate the JAK-STAT pathway [63,64]. In the present study, anti-apoptotic genes were down-regulated, and apoptotic genes were upregulated in AFB1-challenged broiler chicken indicating that excessive apoptosis leads to disturbing the normal function of the spleen. PCPC promoted the secretion of anti-apoptotic proteins (Bcl-2 and Bcl-xl) by regulating the JAK-STAT signaling pathway to a certain extent, thereby promoting the self-renewal of broiler chicken spleen, removing the abnormal cells and preventing excessive apoptosis of spleen cells. PCPC connection with apoptosis finally reversion of the spleen toward a normal state coincides with PCPC effects on the apoptosis pathway in the kidney [29].

This indicated that PCPC could promote the many studies that have highlighted the importance of apoptosis in self-defense mechanisms, the immune system is responsible for protecting the host from a range of external pathogens, apoptosis is a component of the immune system that helps maintain the immune system stability state.

## 5. Conclusions

In this study, PCPC showed an immunoprotective effect on AFB1-induced immune imbalance in broiler chickens that was regulated through JAK/STAT. Activation of the JAK-STAT signaling pathway upregulated the expression of anti-apoptotic gene and apoptotic genes and down-regulated apoptotic genes. PCPC reverses pathological conditions (removal of abnormal cells) in the spleen by preventing excessive apoptosis, and mainly by regulating the JAK/STAT signaling pathway. Finally, this reduced the immune imbalance in broiler chickens with AFB1. By investigating the potential mechanism of immune impairment produced through AFB1 in broiler and the role of the PCPC, we can provide valuable theoretical insights for the practical application of PCPC in social production. However, further research is needed to investigate the mechanisms underlying the immune system imbalance.

## Figures and Tables

**Table 1:** Characteristics of the primers used for qPCR analysis.

Genes	Primer Sequence: (5′-3′)	Product Length (bp)
*Gapdh*	F: CAGAACATCATCCCAGCGTC	20
*Gapdh*	R: GGCAGGTCAGGTCAACAAC	19
*Jak2*	F: GCACAAGCAGAGCATATCGC	20
*Jak2*	R: TCGCCACTGTGCAAATAGGT	20
*Jak3*	F: ATCGCCATCCACGTGTCTAC	20
*Jak3*	R: TCGGGGAAGTCACAGAAGTG	20
*Stat3*	F: GACCAGATGCGAAGGGGTAT	20
*Stat3*	R: CCACAATGCAGGCAATTTGT	21
*Stat5*	F: AGCTGAACGTCACATGAACC	21
*Stat5*	R: TCTCCCCACTGCTCTCATTG	20

## Data Availability

The data presented in this study are available in this article.

## Supplementary Materials

The following supporting information can be downloaded at: https://www.mdpi.com/article/10.3390/vetsci10080521/s1; Table S1:Composition of broiler diet in control group.
