# Supplementary material for: Effect of Penthorum Chinense Pursh Compound on AFB1-Induced Immune Imbalance via JAK/STAT Signaling Pathway in Spleen of Broiler Chicken
Source: Vet Sci. 2023 Aug 13;10(8):521. doi: 10.3390/vetsci10080521 (PMC10459701; doi:10.3390/vetsci10080521)
Supplement: Supplementary file 1 [file vetsci-10-00521-s001.zip › vetsci-2432579-supplementary.pdf]

Table S1 Composition of broiler diet in control group

Ingredients	Proportion（%）
Corn	55.3
Soybean	38.0
Calcium hydrogen phosphate	1.4
Rock flour	1.0
Salt	0.3
Oil	3.0
Additives	1.0
